# Hearing loss in the young-old is associated with increased risk for Alzheimer's disease and vascular dementia

**DOI:** 10.1177/13872877251407211

**Published:** 2026-01-20

**Authors:** Lily Francis, Alexa Beiser, Sophia Lu, Sharon G Kujawa, Nancy Heard-Costa, Francis B Kolo, M Ilyas Kamboh, Rebecca Bernal, D Bradley Welling, Richard L Alcabes, Jayandra J Himali, Sudha Seshadri

**Affiliations:** 1 Glenn Biggs Institute for Alzheimer and Neurodegenerative Diseases, The University of Texas Health Science Center at San Antonio, San Antonio, TX, USA; 2 Department of Neurology, Boston University Chobanian and Avedisian School of Medicine, Boston, MA, USA; 3 Boston University School of Public Health, Boston, MA, USA; 4 Framingham Heart Study, Framingham, MA, USA; 5 Department of Audiology, Harvard Medical School, Massachusetts Eye and Ear Infirmary, Boston, MA, USA; 6 Department of Human Genetics, School of Public Health, University of Pittsburgh, Pittsburgh, PA, USA; 7 Department of Otolaryngology Head and Neck Surgery, Harvard Medical School, Massachusetts Eye and Ear Infirmary, Boston, MA, USA; 8 City of San Antonio, San Antonio, TX, USA; 9 Department of Population Health Sciences, The University of Texas Health Science Center at San Antonio, San Antonio, TX, USA

**Keywords:** Alzheimer's disease, cohort study, dementia, hearing loss, vascular dementia

## Abstract

Hearing loss is a risk factor for dementia, but dementia subtypes underlying this association and effect modifiers are unknown. Using data collected from 2000 Framingham Heart Study participants we found that hearing loss increases risk for Alzheimer's disease and vascular dementia in participants aged 60–70 years (“young-old”) at time of hearing assessment (Alzheimer's disease: HR 1.46[CI 1.07–2.0] p = 0.017; vascular dementia: HR 2.08[CI 1.22–3.56] p = 0.007). Longer duration of hearing loss determines increased risk for Alzheimer's disease and vascular dementia, and screening and intervention for hearing loss from mid-life may help reduce dementia.

## Introduction

Hearing loss, increases with age^1,2^ and is a known risk factor for developing dementia.^3^ The population attributable fraction of age-related hearing loss for dementia is 7%.^4^ However, the type(s) of dementia underlying this association, and modifiers of this association are unknown. We examined whether hearing loss was prospectively associated with an increased risk for all cause dementia, and specifically for Alzheimer's disease (AD) and vascular dementia. We also explored if biological and environmental factors such as age, sex, and use of hearing aids influence this association in the Framingham Heart Study (FHS) Original cohort participants.

## Methods

### Study design, sample, and participants

Enrollment for the FHS Original cohort began in 1948, and participants have been examined biennially since. A total of 2351 participants underwent pure tone audiometry at the 15^th^ biennial exam (1977–1979). The mean age at this exam was 69 years with a range between 57–89 years. We chose participants over the age of 60 years at exam 15, who underwent pure tone audiometry and who had subsequent follow up (up to 40 years) for incident dementia, AD, and vascular dementia. We excluded participants who had a dementia diagnosis at exam 15 (n = 14) or were lost to follow up (n = 172) (Supplemental Figure 1).

### Ascertainment of hearing

Hearing loss was determined by pure tone audiometry administered at exam 15. We defined hearing loss as pure-tone average (0.5, 1.0, 2.0, 4.0 kHz) > 25 dB in the better ear and used the following the National Health and Aging Trends Study (NHATS) categories of better ear pure tone thresholds as follows: normal (0–15 dB), slight hearing loss (range: 16–25 dB), and at least mild hearing loss which is hearing loss with thresholds ≥26 dB and includes moderate, severe and profound hearing loss categories.

### Surveillance for dementia

Our screening and surveillance methods for development of dementia in the Framingham Heart Study have been previously described.^5^ Briefly, all cause dementia was diagnosed using Diagnostic and Statistical Manual of Mental Disorders, fourth edition (DSM-IV) criteria,^6^ AD was diagnosed using the National Institute of Neurological and Communicative Disorders and Stroke and the Alzheimer's Disease and Related Disorders Association (NINCDS–ADRDA) criteria^7^ and the diagnosis of vascular dementia is based on the National Institute of Neurological Disorders and Stroke and the Association Internationale pour la Recherche et l’Enseignement en Neurosciences (NINDS–AIREN) NINDS- AIREN criteria^8^ which are analogous to the most recent VASCOG criteria.^9^

### Statistical analysis

In our primary analysis we used Cox proportional hazards to study time to incident all cause dementia, AD, and vascular dementia across the three levels of hearing (normal or reference group, slight hearing loss, at least mild hearing loss), adjusting for age at exam 15 and sex. The proportional hazards assumption was upheld. The dependent variables were all cause dementia, AD and vascular dementia.

In secondary analysis we examined possible interactions with age and sex. Interactions were considered statistically significant at a p value < 0.1.

We found significant interactions with age, and additionally performed analysis stratified by age. Age was dichotomized using 70 years (median age) for stratified analysis. As a sensitivity analysis, we excluded people who developed dementia within the first three years (Tables 1) which did not change the results.

**Table 1.** Associations between pure tone average (PTA) thresholds and incident dementia stratified by age.

			Age 60–70		Age 70+	
	NHATS categories	p for Interaction	HR (95% CI)	p	HR (95% CI)	p
	All Cause Dementia (n = 633^a^)					
	Normal		1.0		1.0	
	Slight HL	**0**.**02**	1.2 (0.93–1.55)	0.16	0.85 (0.58–1.25)	0.42
	At Least Mild HL		1.56 (1.19–2.05)	**0**.**001**	0.95 (0.67–1.37)	0.79
3-Year Sensitivity Analysis	Normal		1.0		1.0	
	Slight HL		1.18 (0.92–1.53)	0.19	0.88 (0.6–1.31)	0.54
	At Least Mild HL		1.51 (1.14–1.98)	**0**.**004**	0.99 (0.68–1.43)	0.94
	Alzheimer's Disease (n = 491^b^)					
	Normal		1.0		1.0	
	Slight HL	**0**.**04**	1.26 (0.95–1.67)	0.1	0.86 (0.56–1.33)	0.5
	At Least Mild HL		1.46 (1.07–2.0)	**0**.**017**	0.95 (0.63–1.44)	0.82
3-Year Sensitivity Analysis	Normal		1.0		1.0	
	Slight HL		1.26 (0.95–1.67)	0.1	0.89 (0.57–1.39)	0.6
	At Least Mild HL		1.42 (1.04–1.95)	**0**.**03**	0.99 (0.65–1.51)	0.97
	Vascular Dementia (n = 147^c^)					
	Normal		1.0		1.0	
	Slight HL	**0**.**01**	1.28 (0.72–2.26)	0.4	0.68 (0.28–1.63)	0.38

			Age 60–70		Age 70+	
	NHATS categories	p for Interaction	HR (95% CI)	p	HR (95% CI)	p
	At Least Mild HL		1.72 (0.95–3.12)	0.07	1.01 (0.47–2.21)	0.97
3-Year Sensitivity Analysis	Normal		1.0		1.0	
	Slight HL		1.24 (0.70–2.2)	0.47	0.68 (0.28–1.65)	0.39
	At Least Mild HL		1.67 (0.91–3.06)	0.09	0.94 (0.42–2.07)	0.87

^a^ 354/633 participants (56%) were between the ages of 60–70 years.

^b^ 279/491 participants (57%) were between the ages of 60–70 years.

^c^ 89/147 participants (61%) were between the ages of 60–70 years.

Adjusted for age at exam 15 and sex.

HR: Hazard Ratio; CI: Confidence Interval.

Associations were considered statistically significant at p < 0.05. Interactions were considered statistically significant at p < 0.1.

Bold type indicates a p value less than 0.05 or an interaction p value less than 0.1.

Since we observed differences in education and cardiovascular disease prevalence between hearing groups, we adjusted for education and Framingham Stroke Risk Profile^10^ (FSRP) in additional models but failed to observe significant changes in the strength of associations (data not shown).

All statistical analysis was performed using SAS statistical software version 9.4 (SAS Institute). These study protocols and consent forms were approved by the Institutional Review Board of Boston University Medical Center. All participants provided written informed consent before study commencement. The study was performed in accordance with guidelines of the Institutional Review Board of University of Texas Health Science Center at San Antonio.

## Results

The final sample consisted of 2000 people from the original cohort, of whom 60% were female (Table 2). The mean age of our participants at exam 15 was 69.5 years (IQR 63.9–73.9).

**Table 2.** Participant characteristics at baseline (original cohort, exam 15, 1977–1979).

Baseline characteristics	Overall (n = 2000)	Normal Hearing (n = 536)	Mild Hearing Loss (n = 593)	At least Moderate Hearing Loss (n = 871)
Age, mean (range), y	69.5 (60–88.7)	66.36 (60–84.1)	68.11(60.1–85.6)	72.48 (60.1–88.7)
Women, n (%)	1206 (60.3)	408 (76.1)	366 (61.7)	432 (49.6)
Educational level, n (%)				
No High School Diploma	749 (38)	150 (28.4)	219 (37.5)	380 (44.3)
High School Diploma	629 (32)	190 (36)	181 (31)	258 (30.1)
Some College	355 (18)	113 (21.4)	111 (19)	131 (15.3)
College Degree and Above	236 (12)	75 (14.2)	73 (12.5)	88 (10.3)
Hearing aid use, n (%)	114 (5.7)	none	1 (0.05)	113 (13)
Blood pressure, mean (SD), mm Hg				
Systolic	136.6 (18.7)	134.76 (19)	135.88 (18.8)	138.23 (18.3)
Diastolic	75.66 (10.1)	75.77 (10.2)	75.73 (9.7)	75.54 (10.3)
Antihypertensive medication use, n (%)	669 (33.6)	170 (31.8)	195 (33.1)	304 (35)
Prevalent cardiovascular disease, n (%)	461 (23.1)	85 (15.9)	127 (21.4)	249 (28.6)
BMI, mean (SD), kg/m^2^	26.49 (4.3)	26.49 (4.3)	26.66 (4.5)	26.37 (4.1)
Total Cholesterol, mean (SD), (mg/dL)	230.47 (41)	236.16 (40.9)	231.87 (41.4)	226.02 (40.3)
HDL, mean (SD), (mg/dL)	49.82 (15.7)	53.28 (15.8)	50.11 (15.9)	47.49 (15.1)
Diabetes, n (%)	183 (9.5)	35 (6.8)	56 (9.8)	92 (11)
Current smoking, n (%)	396 (20.3)	112 (21.3)	129 (22.2)	155 (18.3)

BMI: body mass index; HDL: high density cholesterol.

We found that at least mild hearing loss was associated with increased risk for all cause dementia, AD and vascular dementia, while we did not identify significant associations with slight hearing loss. Since we found a significant interaction with age for all cause dementia, AD and vascular dementia, we performed age stratified analyses. People with at least mild hearing loss who were between 60–70 years at biennial exam 15 had a 56% (HR 1.56 [CI1.19–2.05] p = 0.001) greater risk of subsequent all cause dementia, 46% (HR 1.46[CI1.07–2.0] p = 0.017) greater risk of AD and 108% (HR 2.08[CI1.22–3.56] p = 0.007) greater risk of vascular dementia (Table 1). No such increased risk was observed in participants older than 70 years (all cause dementia: HR 0.95[CI 0.67–1.37] p = 0.79; AD: HR 0.95[CI 0.63–1.44] p = 0.82; Vascular dementia: HR 1.01[CI0.47–2.21] p = 0.97) at the time of hearing evaluation. As a secondary analysis we included all participants with pure tone average (PTA) at exam 15 (age range 57–88) and found that people with at least mild hearing loss who were between 55–70 years had similar increases in risk for dementia as compared to the 60–70-year age group (data not shown).

**Figure 1.**
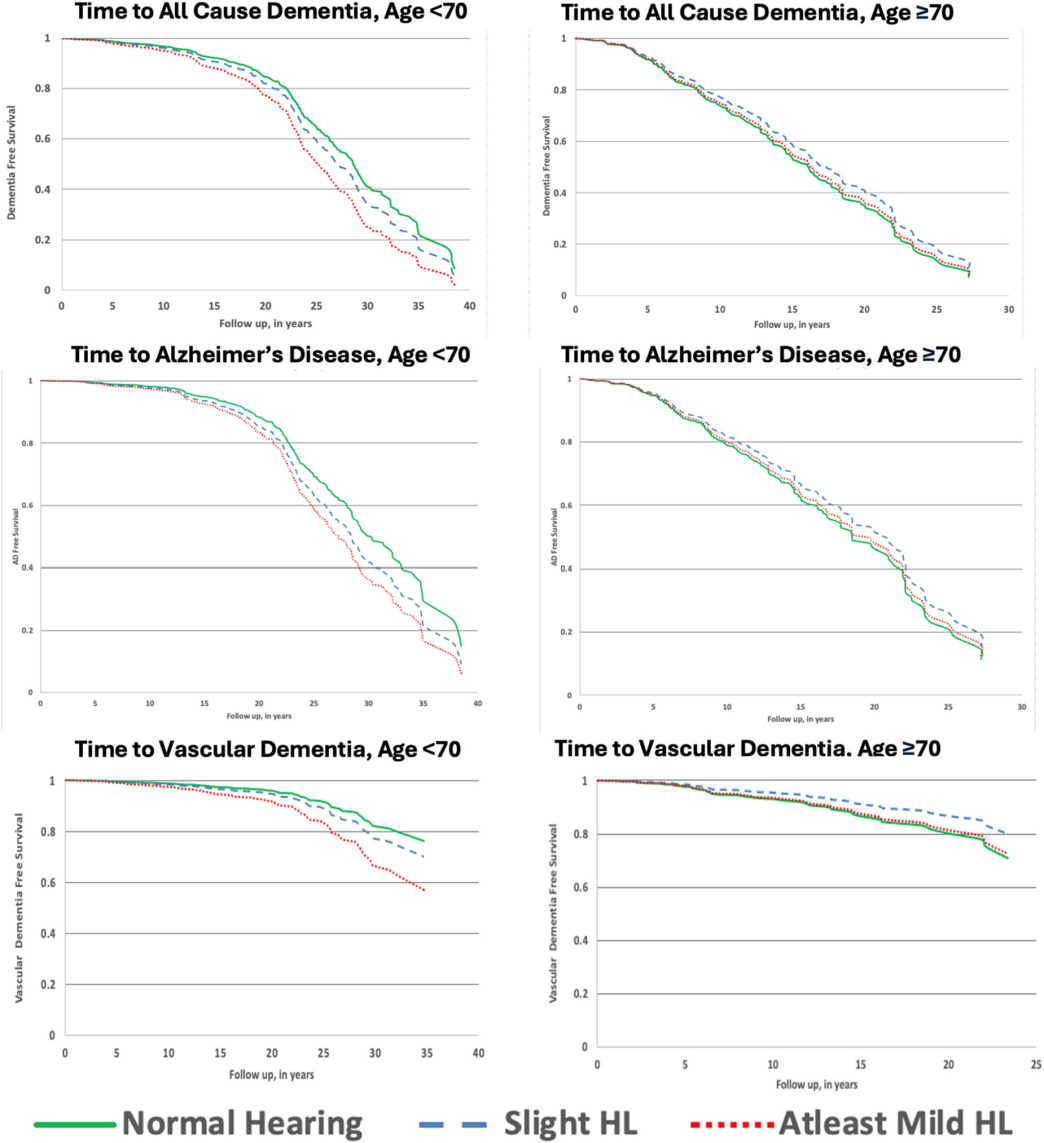
Kaplan-Meier survival curves showing time to dementia stratified by age groups <70y and ≥70y at the time of hearing evaluation.

We constructed Kaplan-Meier survival curves for time to dementia stratified into two groups by age at hearing evaluation (Age <70, Age ≥70) and observed a decreased time to dementia in the Age <70 group commensurate with severity of hearing loss (Figure 1).

### Contribution of other factors (hearing aids, sex) to association between hearing loss and dementia risk

In our sample 13% of people with hearing loss used hearing aids and we did not have sufficient power to test for interaction or conduct a subgroup analysis. When we ran a model adjusting for hearing aid usage we failed to see significant changes in the strength of the associations. However, the limited usage and late initiation of hearing aids significantly constrain interpretation of the finding that hearing aid use did not modify dementia risk. Use of hearing aids starting at a younger age would likely lead to dementia risk reduction.^11^ We found no significant interaction with sex.

## Discussion

### Association of hearing loss with AD, vascular dementia, and all cause dementia

We have reported that hearing loss is associated with increased risk for both AD and vascular dementia. Other longitudinal cohort studies utilizing pure tone audiometry to measure hearing have reported an increased risk of incident dementia in people with hearing loss,^3,12,13^ although they did not identify independent associations with AD and vascular dementia or on age stratified results. A recent meta-analysis of studies examining the relationship of objectively measured hearing loss to dementia reported a 28% increased risk of dementia in people with hearing loss although it also failed to uncover an association with AD.^14^ However a retrospective study of health conditions associated with AD and vascular dementia identified hearing loss as associated with both conditions.^15^

### The interaction between hearing loss and age at hearing evaluation in the association with subsequent dementia

We found that at least mild hearing loss increases risk of AD, vascular dementia, and all cause dementia only in people who were younger than 70 years at the time of hearing evaluation. Other studies on life course illnesses and dementia risk have reported that midlife vascular risk factors^16,17^ mid-life stressors^18^ and midlife cardiovascular health^19^ are more strongly associated with development of late onset dementia than late life exposure to these risk factors. Our study suggests that the association of hearing loss with increased risk of AD and vascular dementia depends on both the duration and severity of hearing loss such that we see increased risk only in those participants with at least mild hearing loss who were evaluated for hearing loss before the age of 70 years. We propose that age related hearing loss should also be viewed from a life course perspective, warranting screening and management during mid-life to prevent late onset dementia.

### Are hearing aids beneficial in reducing risk of dementia?

We found that self-reported use of hearing aids did not significantly alter the risk for subsequent dementia. However, in our sample of participants with at least mild hearing loss, only 13% used hearing aids, and the median age of participants when they were acquired was 73.7 years. This may explain the lack of dementia risk reduction from hearing aid usage. A recent randomized controlled trial of hearing aids showed no change in the 3 -year cognitive decline in the total cohort, but observed that hearing aids may benefit a subset of the population at high risk for developing dementia.^20^ Findings from a recent meta-analysis demonstrated a 17% lower hazard for incident dementia by use of hearing aids.^21^

### How does hearing loss result in increased risk of Alzheimer's disease and vascular dementia?

Hearing loss could increase dementia risk by causal mechanisms such as increasing social isolation, increasing the cognitive load or directly affecting auditory areas of the brain. An alternate hypothesis is that hearing loss and increased dementia risk could both result from a common underlying mechanism like microvascular disease.^22^ Causal mechanisms leading to increased dementia risk can be corrected by hearing intervention, while common mechanisms would show no benefit.^23^ The larger effect sizes that we see for vascular dementia suggest that both causal and common mechanisms may be at play in the association of hearing loss with increased risk for incident vascular dementia. Alternatively, hearing loss may reflect broader neurodegenerative or vascular pathology already underway.

### Strengths and limitations

Strengths of this study are the large sample size, standardized hearing assessment by pure tone audiometry, and the long period of rigorous dementia follow up for up to four decades following hearing evaluation. There are also limitations: we have not directly explored causality in the association between hearing loss and dementia, although the timing of hearing measurement predates dementia diagnosis by up to 40 years, and when in a sensitivity analysis we excluded people who developed dementia in the first three years our results did not change significantly (Tables 1). We examined AD and vascular dementia as individual entities, although studies indicate that mixed AD and cerebrovascular disease is the most common pathologic dementia diagnosis,^24^ and that up to 30% of clinical AD cases can be attributed to vascular pathologies on autopsy.^25^ The criteria used for clinical diagnosis of AD and vascular dementia have since been updated.^26^ A comprehensive re-review of dementia cases using up to date criteria was carried out at Framingham beginning in 2001, and a high degree of consistency has been reported between a clinical diagnosis of AD using these criteria and the subsequent neuropathological confirmation of AD among brain donors in FHS.^27^ The Framingham Original cohort is overwhelmingly Caucasian. Different populations have different susceptibility to environmental^28^ and genetic risk factors^29^ for dementia, and these findings must be replicated in participants from other races and genetic ancestries for generalizability.

### Public health implications

We have shown an association between hearing loss and incident AD, vascular dementia and all cause dementia is restricted to FHS participants younger than 70 years at time of hearing assessment. This suggests that the duration of hearing loss impacts the increased risk for AD and vascular dementia. Hearing loss was identified as the strongest modifiable risk factor for dementia risk reduction by the Lancet Commission, with a potential to eliminate 7% of dementia cases by treating hearing loss.^4^ The ACHIEVE Randomized Controlled Trial showed that hearing aids reduced the risk for cognitive decline by 48% over 3 years in older high-risk individuals,^20^ and our recent observational study shows that use of hearing aids in the young-old was associated with a 61% reduction in risk for dementia.^11^ Yet only 14% of individuals with hearing loss use hearing aids, with up to 23 million older Americans experiencing untreated hearing loss.^30^ Medicare neither covers the cost of exams for fitting hearing aids, nor the cost of hearing aids. Our findings have important public health implications that could include policy changes facilitating early screening and management of hearing loss for optimal dementia risk reduction.

## Supplemental Material

sj-docx-1-alz-10.1177_13872877251407211 - Supplemental material for Hearing loss in the young-old is associated with increased risk for Alzheimer's disease and vascular dementia Supplemental material, sj-docx-1-alz-10.1177_13872877251407211 for Hearing loss in the young-old is associated with increased risk for Alzheimer's disease and vascular dementia by Lily Francis, Alexa Beiser, Sophia Lu, Sharon G Kujawa, Nancy Heard-Costa, Francis B Kolo, M Ilyas Kamboh, Rebecca Bernal, D Bradley Welling, Richard L Alcabes, Jayandra J Himali and Sudha Seshadri in Journal of Alzheimer's Disease
