# Supplementary material for: Hearing loss in the young-old is associated with increased risk for Alzheimer's disease and vascular dementia
Source: J Alzheimers Dis. 2026 Jan 20;109(3):1142–8. doi: 10.1177/13872877251407211 (PMC12855611; doi:10.1177/13872877251407211)
Supplement: sj-docx-1-alz-10.1177_13872877251407211 - Supplemental material for Hearing loss in the young-old is associated with increased risk for Alzheimer's disease and vascular dementia [file sj-docx-1-alz-10.1177_13872877251407211.docx]

**Supplemental Figure 1**


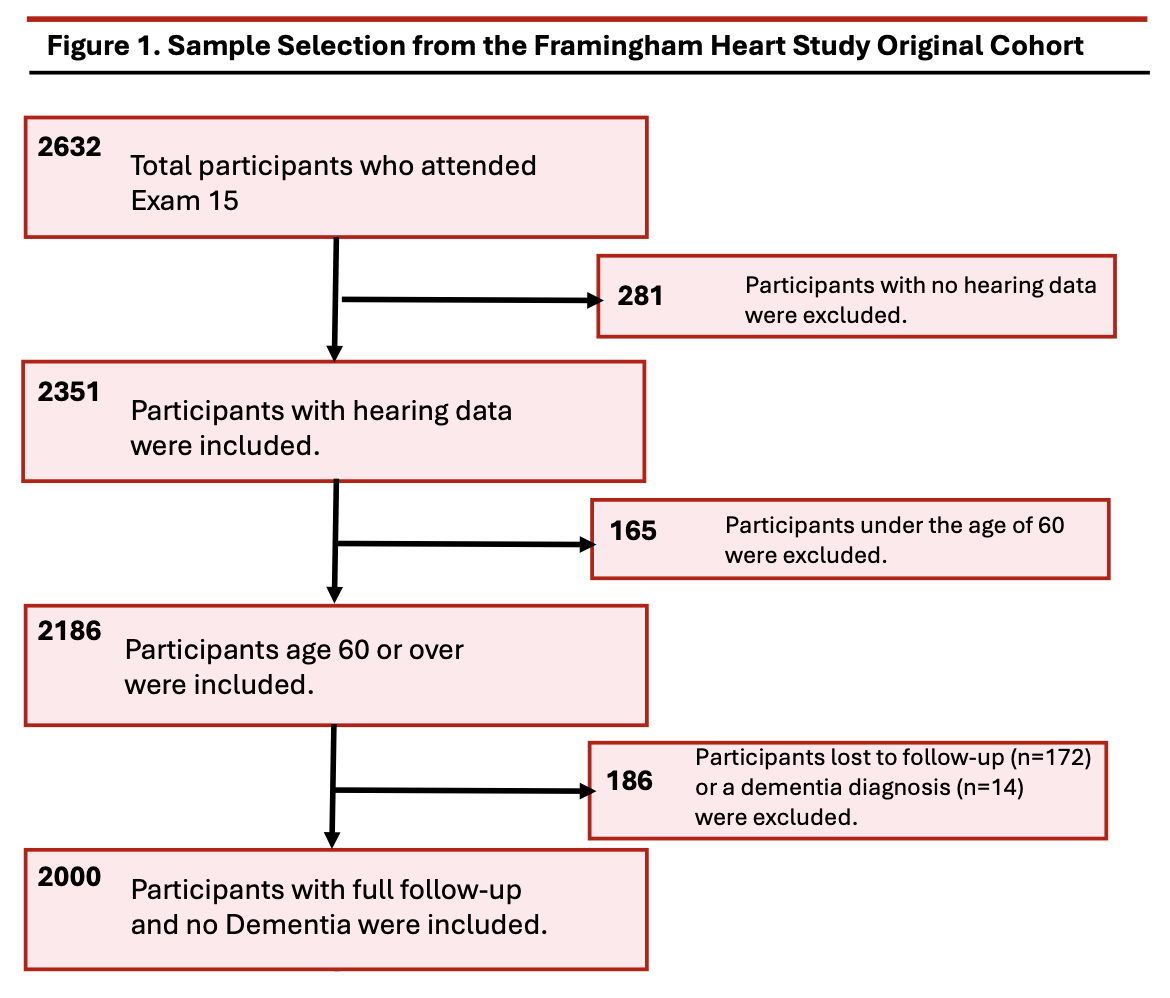


The baseline examination period was between 1977 and 1979. Participants who had no hearing data, had preexisting dementia, younger than 60y of age, or had incomplete follow up were excluded.
